# Insight Into the Distribution of Staphylococci and Their Enterotoxins in Cheeses Under Natural Conditions

**DOI:** 10.3389/fmicb.2018.03233

**Published:** 2019-01-07

**Authors:** Alberto Bellio, Francesco Chiesa, Silvia Gallina, Daniela Manila Bianchi, Guerrino Macori, Dario Bossi, Yacine Nia, Isabelle Mutel, Sabine Messio, Jacques-Antoine Hennekinne, Lucia Decastelli

**Affiliations:** ^1^National Reference Laboratory for Coagulase Positive Staphylococci, Istituto Zooprofilattico Sperimentale del Piemonte, Liguria e Valle d’Aosta, Turin, Italy; ^2^Dipartimento di Medicina Veterinaria, Università degli Studi di Torino, Turin, Italy; ^3^S.C. Controllo Alimenti e Igiene delle Produzioni, Istituto Zooprofilattico Sperimentale del Piemonte, Liguria e Valle d’Aosta, Turin, Italy; ^4^Servizio Veterinario Asl VC, Vercelli, Italy; ^5^European Union Reference Laboratory for Coagulase Positive Staphylococci, Agence Nationale de Sécurité Sanitaire de l’Alimentation, de l’Environnement et du Travail, Maisons-Alfort, France

**Keywords:** *Staphylococcus aureus*, enterotoxins (SEs), foodborne, cheese, raw milk

## Abstract

Staphylococcal food poisoning outbreaks are a major cause of food-borne illness in the European Union and their notification has been mandatory since 2005. Criteria for the enumeration of coagulase-positive Staphylococci (CPS) and the detection of staphylococcal enterotoxins (SEs) in cheese have been set down in Commission Regulation EC 2073/2005. Currently, few information are available about the distribution of SEs in naturally contaminated cheeses, including raw-milk and artisanal dairy products. The aim of this study was therefore to investigate at both the CPS enumeration and the succession of the enterotoxigenic *Staphylococcus aureus* and produced enterotoxins levels on the rind and the core of a raw-milk semi-hard cheese, produced on farm. The study has been conducted in three steps: (I) seven wheels at different time of ripening where tested for the presence of SEs. (II) from each wheel, four portions were subsequently sampled from four different areas (peripheral rind, central rind, peripheral core and central core). (III) two cheese wheels, characterized by the highest and lowest CPS numbers and SEs quantification, based on the second step of the study, were further analyzed. A significant difference has been observed in the distribution of CPS and SEs in the four areas sampled, irrespectively of the batch and the time of ripening. The results of this study provided a set of previously unknown information on the influence of natural conditions on the distribution of CPS and SEs thereof in the cheese matrix, filling a gap in the understanding of SEs biosynthesis process.

## Introduction

Staphylococcal food poisoning outbreaks are a major cause of food-borne illness in the European Union and their notification has been mandatory since 2005 (European Food Safety Authority [EFSA] and European Centre for Disease Prevention and Control [ECDC], 2015). Criteria for the enumeration of coagulase-positive Staphylococci (CPS) and the detection of staphylococcal enterotoxins (SEs) in cheese have been set down in Commission Regulation EC 2073/2005 (European Commission [EC], 2005) amended by Commission Regulation (EC) 1441/2007 (European Commission [EC], 2007). This regulation defines process hygiene criteria and food safety criteria. For milk and milk products, SEs detection must be performed when the CPS count exceeds 10ˆ5 colony forming units per gram (cfu/g) (Bianchi et al., 2014; Nia et al., 2016). In fact, in cheese, this is considered a favorable condition for the production of enterotoxins and, as a result of the heat-stable nature of the SEs, they may be present where viable *Staphylococcus aureus* are absent (Jablonski and Bohach, 1997; Le Loir et al., 2003). The diversity of cheese production reflects its uniqueness in terms of taste, flavoring and textures (Macori and Cotter, 2018). Some productions can follow traditional processes and recipes, including the use of raw milk that has been reported as the responsible source that caused foodborne outbreaks (Jørgensen et al., 2005; Johler et al., 2015, 2018; Armani et al., 2016), concur with contamination in different critical stage of the production pipelines (Kümmel et al., 2016; McHugh et al., 2017). The growth of *S. aureus* and the production of enterotoxins are also influenced by environmental conditions such as temperature, pH, water activity, salt concentration (Sihto et al., 2014; Beuchat, 2017) and cheese microbiota, competing and affecting its virulence (Schelin et al., 2011; Bueno et al., 2012; Bonham et al., 2017; Cotter et al., 2017). Cheese microbiota has been recently used as a model for describing the formation and function of microbial communities (Wolfe et al., 2014) and for better understand the role of individual species in the rind of surface-ripened cheese (Bertuzzi et al., 2018). The microbial distribution in cheeses can be spatially affected by the ripening and different studies investigated its evolution over time, especially starters and non-starter lactic acid bacteria (NSLAB) (Fitzsimons et al., 2001; Levante et al., 2017). Currently, few information are available about the distribution of SEs in naturally contaminated cheeses, including raw-milk and artisanal dairy products. The aim of the current study was to investigate the distribution and quantification of CPS and produced enterotoxins on the rind and the core of a raw-milk semi-hard cheese, produced on farm. Studies were performed on batches of cheeses sampled over the course of 10 weeks of ripening to correlate surfaces and cores bacterial dynamics in order to understand how CPS contamination levels and spatial distribution relates to SEs diffusion and concentrations.

## Materials and Methods

### Natural-Contaminated Samples

Seven wheels of naturally contaminated cheese matrices, representing seven different batches at different ripening time (4 – 10 weeks) were identified during own check analysis performed by a local producer in Italy and conferred to the Italian National Reference Laboratory for Coagulase Positive Staphylococci (NRL CPS). The cheeses studied were a local type of “toma cheese,” a semi-hard cheese from raw cows’ milk, produced on farm. After milking, milk was immediately cooled to not more than 6°C and processed every 48 h; the procedure for cheese making is as follows: the milk is heated to 35°C; rennet is added and the curd is heated to 39°C (semi-cooked) for 60 min, lightly pressed into the mold and left draining at room temperature for approximately 12 h. The characteristics of the seven batches are reported in Table 1.

**Table 1:** Size and ripening of the seven cheese wheels included in this study.

No Batch	Weight (kg)	Diameter (cm)	Height (cm)	Ripening time (weeks)
1	1.7	22	5.5	4
2	1.7	22	5.5	5
3	1.7	22	5.5	6
4	1.7	22	5.5	7
5	1.7	22	5.5	8
6	1.7	22	5.5	9
7	1.4	17	6.0	10

### Experimental Design

**FIGURE 1:**
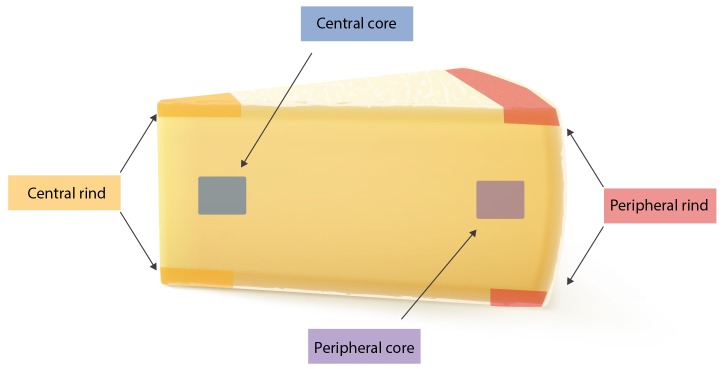
Sampling location.

The study has been conducted in three steps: (I) 25 g from each of the seven wheels where tested for the presence of SEs using both Vidas SET2 detection kit (bioMerieux, Marcy-l’Étoile, France) and Ridascreen SET Total (R-Biopharm, Darmstadt, Germany). In-house developed quantitative ELISA method was then used to confirm and quantify the SEs; (II) from each wheel, four portions of 25 g were subsequently sampled from four different areas (peripheral rind, central rind, peripheral core and central core) (Figure 1). In total, 28 samples were collected and submitted to CPS enumeration and SEA and SED quantification. (III) two cheese wheels, batch 4 and 7, characterized, respectively, by the highest and lowest CPS numbers and SEs quantification, based on the second step of the study, were further analyzed. From each wheel, ten slices were sampled (130 g) and sub-sampled as previously described. In total, 40 samples were collected from each wheel and tested as described in the second step. The cheese rinds were aseptically removed using a sterile knife and the core sampled using a sterile cheese trier after all the rind had been removed.

### Enterotoxins Detection

Twenty five gram of samples (cores and rinds) were analyzed for the presence of SEs according to the European Screening Method (ESM) of the European Reference Laboratory for Coagulase Positive Staphylococci (EU RL CPS), consisting of extraction followed by a dialysis concentration. The staphylococcal enterotoxins where detected by immunological detection using both the methods previously described. With these methods, the enterotoxins A, B, C, D and E are simultaneously detected in dairy products. For VIDAS, the results are expressed as TV, which is the ratio between the relative fluorescence value (RFV) of the solution tested and the RFV of the standard in the VIDAS SET2 kit. A TV value ≥0.13 was considered positive. Characterization and quantification of SEs were performed by a quantitative indirect sandwich-type ELISA. A single sandwich type was used for SEB whereas double sandwich ELISA types were used for SEA, SEC and SED. For the detection step, specific commercially available antibodies (Toxin Technology, Sarasota, Florida, United States) were used as coating (ref SLAI1 01, SLBI202, SLCI11, SLDI303 for SEA, SEB, SEC and SED, respectively) and probing antibodies (ref LAI101, LCI 111, and LDI303 for SEA, SEC and SED, respectively). The presence of toxins was revealed by immunoglobulins coupled to horseradish peroxidase (ref LBC202 for SEB and goat-anti rabbit coupled to peroxidase antibodies for SEA, SEC and SED, KPL) and determined by a colorimetric measurement at 405/630 nm after addition of ABTS (KPL) (Hennekinne et al., 2012). All statistical analysis was executed using GraphPad Prism 7.0 software (GraphPad, San Diego, CA, United States).

### Plate Counting of CPS

Coagulase-positive Staphylococci were counted in samples using the standard method EN ISO 6888 part 2 (International Standardization for Organization [ISO], 1999) at the Italian National Reference Laboratory for CPS, including *S. aureus* (ITNRL-CPS) in Turin, Italy. Briefly: 10 g of the sampled cheese was added to 9 parts of buffered peptone water (90 ml) and mixed. 1 ml was then pour-plated with freshly prepared Rabbit plasma fibrinogen agar medium, after appropriate decimal dilutions and incubated at 37°C for 24 h.

### Typing and Characterization of the Isolates by Multiplex PCR

Two colonies, isolated from counting plates from each sub-sampled portion in the second step of the study, were subjected to SEs genes characterization, through two multiplex PCR protocols used according to European Union Reference Laboratory for Coagulase-Positive Staphylococci (EU-RL CPS) methods (Kérouanton et al., 2007), and biotyped as described by Devriese (1984).

## Results

### Population Structure

**FIGURE 2:**
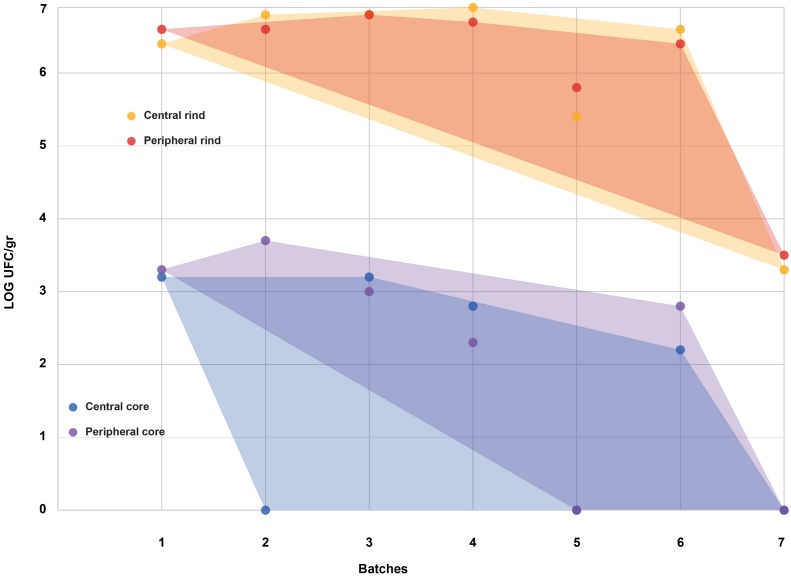
Coagulase-positive Staphylococci (CPS) counts according to the four sampled areas (step II). The smallest convex shape containing the points of a single area was added.

**FIGURE 3:**
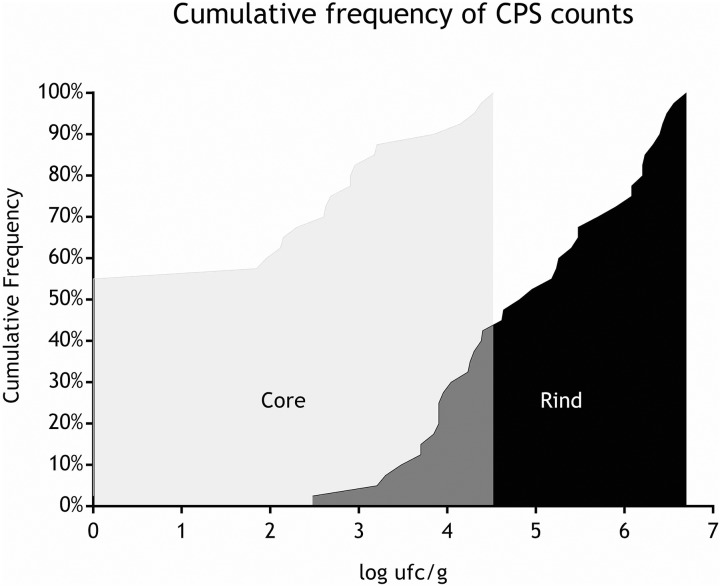
Cumulative frequency of CPS counts (step II).

All the batches, analyzed in the first step, showed the presence of CPS, with counts ranging from 3,52 to 5 Log cfu/g (Table 2). In 3 sub-samples, out of the 28 obtained in the second part of the study, CPS count was <1 Log cfu/g. The presence of CPS, in the second step of the study, was observed in 82% (*n* = 23) of the samples analyzed (*n* = 28), with a concentration ranged between values <1 and 3,7 Log cfu/g in the core samples and between 3,3 and 6,9 Log cfu/g in the rind samples (Table 3). CPS counts from the four different areas (peripheral rind, central rind, peripheral core and central core) in the second and third steps demonstrated a significant difference between rind and core numbers of CPS (Kruskal–Wallis test, *p* < 0,001) with a higher contamination in the rind and lower contamination in the core (Figures 2, 3), while the center versus periphery effect was not significant (data not shown). In Figure 3 cumulative frequency was calculated and plotted against the average logarithmic values to show the difference between the two areas.

**Table 2:** Coagulase-positive Staphylococci enumeration and SEs detection and quantification in the first step of the study.

No. batch	Log cfu/g	Result of ESM		Quantification	
		Vidas (TV)	Ridascreen (AU)	SEA ng/g	SED ng/g
1	5	2.70	3.957	0.721	1.524
2	5	2.82	3.778	0.262	0.474
3	5	2.52	3.956	1.538	2.414
4	4.08	2.35	3.957	1.784	3.187
5	4.36	3.01	3.961	0.175	1.503
6	3.52	2.90	3.835	0.151	1.071
7	3.52	0.85	0.623	0.020	< LoD^∗^

*^∗^Limit of detection estimated at 0.013 ng/g.*

**Table 3:** Coagulase-positive Staphylococci enumeration and SEs detection and quantification in the second step of the study.

Batch	Sampling location	Log UFC/g	Vidas (TV)	SEA (ng/g)	SED^∗^ (ng/g)
1	Central core	3.2	2.25	0.90	2.28
	Peripheral core	3.3	2.43	0.30	0.60
	Central rind	6.4	2.16	0.68	1.89
	Peripheral rind	6.6	1.17	0.39	1.57
2	Central core	1.5	2.40	0.26	0.61
	Peripheral core	3.7	1.29	0.35	0.74
	Central rind	6.8	1.75	0.62	1.87
	Peripheral rind	6.6	1.24	0.30	4.12
3	Central core	3.2	3.97	2.71	4.64
	Peripheral core	3.0	3.04	1.51	2.31
	Central rind	6.8	2.42	1.93	4.07
	Peripheral rind	6.8	2.60	1.00	4.41
4	Central core	2.8	3.02	1.85	3.67
	Peripheral core	2.3	2.99	1.27	2.92
	Central rind	6.9	3.01	1.65	4.97
	Peripheral rind	6.7	2.99	1.80	8.34
5	Central core	< 10 cfu/g	3.45	0.64	3.14
	Peripheral core	1.5	0.62	1.79	8.98
	Central rind	5.4	2.73	0.62	2.44
	Peripheral rind	5.8	3.21	0.85	3.59
6	Central core	2.2	3.54	0.58	1.47
	Peripheral core	2.8	3.02	0.54	1.30
	Central rind	6.6	2.79	0.55	2.22
	Peripheral rind	6.4	2.65	0.41	2.07
7	Central core	< 10 cfu/g	1.09	0.01	< LoQ
	Peripheral core	< 10 cfu/g	0.38	0.03	0.09
	Central rind	3.3	0.25	0.01	< LoQ
	Peripheral rind	3.5	1.17	0.05	0.06

*^∗^LOQ for SED was estimated at 0.035 ng/g.*

From a total of 50 isolates, 41 showed the presence of at least one SE gene, with the most prevalent SE genes profile being *sea-sed-sej-ser*, representing the 68% of all the isolates. The human biotype demonstrated to be the most common in the population studied (Table 4).

**Table 4:** Profiles obtained combining biotyping and SEs genes presence.

Profile	Biotype	SEs genes	Isolates
1	Human	*seh*	2
2	Human	*sej*	1
3	Human	*sea-sed-ser*	3
4	Human	*sea-sed-sej-ser*	25
5	Human	neg	2
6	NHS3	*sea-sed-sej-ser*	8
7	NHS3	neg	6
8	NHS5	*sea-sed-sej-ser*	1
9	NHS5	neg	1
10	NHS6	*sea-sed-seh-sej-ser*	1

### Enterotoxins

Limits of detection (LOD) and of quantification (LOQ) were estimated at 0.003 and 0.01 ng/g for SEA, and at 0.013 and 0.035 ng/g for SED, respectively.

The results obtained, in the first part of the study, by the ESM method using both Vidas SET2 and Ridascreen SET Total kits showed the presence of SE in all the studied cheeses. Results obtained by the quantitative ELISA method confirmed the SEs contamination and indicated the presence of two SE types, SEA and SED and therefore only these two SEs were targeted in the following steps of the study. The results are summarized in Table 2.

The results obtained in the second step of the study are shown in Table 3. The quantitative ELISA method confirmed the high concentrations of SEA and SED for six wheels (No. 1–6). In the case of wheel No. 7, both SEA and SED were detected in contrast to the results obtained in the first step where only SEA was detected in this wheel. However, the wheel No. 7 appeared to be contaminated by, SEA and SED, at low concentration level compared to the other wheels.

**FIGURE 4:**
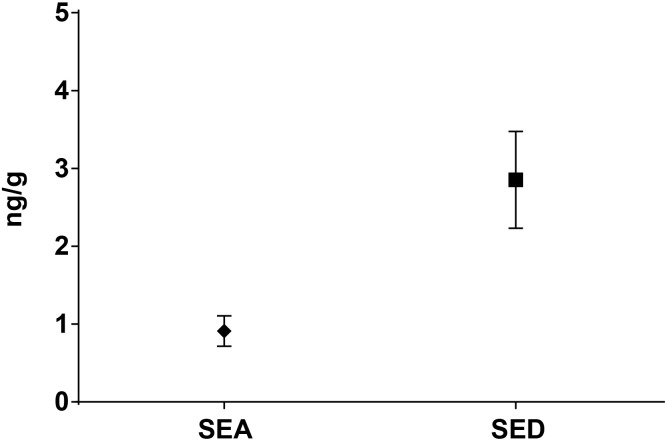
Means difference of SEA and SED quantification (step II). Means and standard deviation are showed.

**FIGURE 5:**
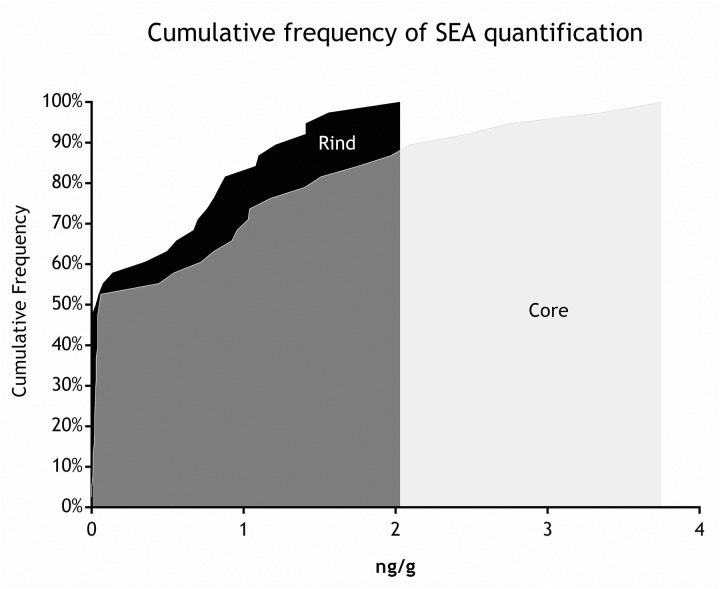
Cumulative frequency of SEA quantification (step III).

**FIGURE 6:**
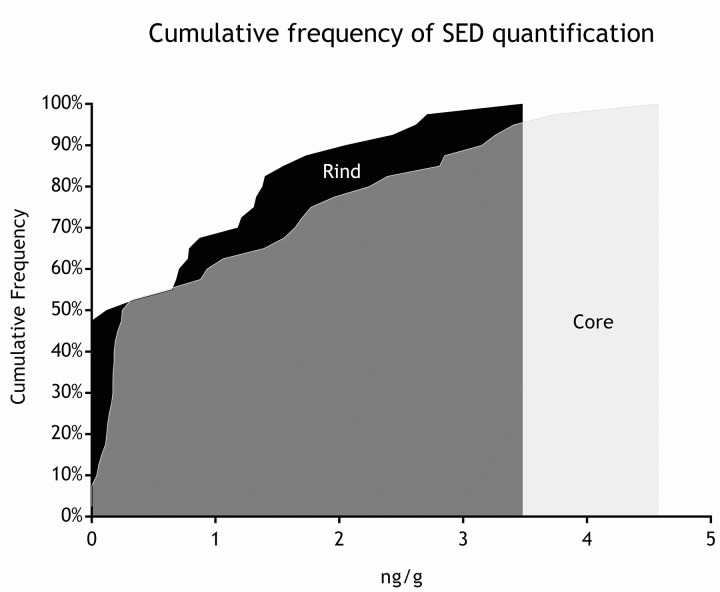
Cumulative frequency of SED quantification (step III).

As for SEs quantification, while SED concentrations were significantly higher than SEA concentrations, whatever the wheel or the sampling area studied (Wilcoxon test, *p* < 0,0001) (Figure 4 and Tables 5, 6), the analysis performed in the third step of the study demonstrated a higher contamination in core (Mann–Whitney test, *p* < 0,05) for both SEA and SED whatever the wheel sampled. In Figures 5, 6 cumulative frequency was calculated and plotted against the average quantification values to show the difference between the two areas.

**Table 5:** Batch N°4 -SEs detection and quantification in the third step of the study.

Sub-sample ID	Sampling location	Vidas (TV)	SEA^∗^ (ng/g)	SED (ng/g)
1	Central core	3.35	3.75	4.58
	Peripheral core	3.7	2.75	3.15
	Central rind	1.55	0.84	0.78
	Peripheral rind	3.14	1.41	0.87
2	Central core	3.52	1.75	3.26
	Peripheral core	4.21	0.96	1.39
	Central rind	2.27	0.88	1.18
	Peripheral rind	0.67	0.35	1.33
3	Central core	3.29	1.51	1.96
	Peripheral core	3.57	1.04	1.64
	Central rind	2.13	0.56	0.35
	Peripheral rind	2.9	0.50	0.71
4	Central core	3.06	2.47	3.41
	Peripheral core	3.55	1.97	2.24
	Central rind	2.28	2.03	2.62
	Peripheral rind	1.84	1.21	2.71
5	Central core	4.12	1.40	1.70
	Peripheral core	4.2	0.72	1.06
	Central rind	2.29	1.41	1.38
	Peripheral rind	2.59	0.81	2.05
6	Central core	3.33	3.35	3.74
	Peripheral core	3.98	2.09	2.39
	Central rind	2.74	1.08	1.73
	Peripheral rind	2.43	0.70	2.43
7	Central core	4.21	1.03	1.55
	Peripheral core	4.21	0.92	0.88
	Central rind	2.34	1.56	1.40
	Peripheral rind	1.99	0.76	0.79
8	Central core	3.18	0.54	0.60
	Peripheral core	4.21	0.80	0.93
	Central rind	3.83	1.10	1.55
	Peripheral rind	2.11	0.67	1.21
9	Central core	4.21	1.18	1.77
	Peripheral core	2.67	0.44	0.32
	Central rind	1.36	< LOD	0.65
	Peripheral rind	2.63	0.08	1.31
10	Central core	4.05	^∗∗^	2.81
	Peripheral core	3.9	^∗∗^	2.85
	Central rind	2.05	0.60	0.68
	Peripheral rind	4.01	0.14	3.48

*^∗^LOD for SEA was estimated at 0.003 ng/g. ^∗∗^uninterpretable results.*

**Table 6:** Batch N°7 -SEs detection and quantification in the third step of the study.

Sub-sample ID	Sampling location	Vidas (TV)	SEA^∗^ (ng/g)	SED^∗∗^ (ng/g)
1	Central core	1.7	0.04	0.14
	Peripheral core	1.3	0.02	0.08
	Central rind	0.59	< LOQ	< LOD
	Peripheral rind	0.47	< LOQ	< LOD
2	Central core	2.01	0.03	0.21
	Peripheral core	1.19	0.01	0.04
	Central rind	0.56	< LOD	< LOD
	Peripheral rind	0.67	< LOQ	< LOD
3	Central core	1.81	0.03	0.16
	Peripheral core	0.94	< LOQ	< LOQ
	Central rind	0.68	< LOD	< LOD
	Peripheral rind	0.47	< LOD	< LOD
4	Central core	1.36	0.03	0.17
	Peripheral core	0.62	0.01	< LOQ
	Central rind	0.23	< LOD	< LOD
	Peripheral rind	0.29	< LOQ	< LOD
5	Central core	1.69	0.02	0.17
	Peripheral core	0.31	< LOQ	< LOQ
	Central rind	0.2	< LOD	< LOD
	Peripheral rind	0.36	< LOQ	< LOD
6	Central core	1.42	0.04	0.18
	Peripheral core	1.5	0.04	0.18
	Central rind	0.19	< LOD	< LOQ
	Peripheral rind	0.39	< LOQ	< LOD
7	Central core	1.6	0.05	0.24
	Peripheral core	1.64	0.06	0.25
	Central rind	0.52	< LOQ	< LOQ
	Peripheral rind	0.83	0.03	< LOD
8	Central core	1.26	0.03	0.13
	Peripheral core	1.44	0.02	0.18
	Central rind	0.69	< LOD	0.12
	Peripheral rind	0.9	0.05	< LOD
9	Central core	1.43	0.04	0.19
	Peripheral core	1.53	< LOD	0.06
	Central rind	0.47	< LOD	< LOD
	Peripheral rind	0.47	< LOD	< LOD
10	Central core	1.36	0.03	0.12
	Peripheral core	1.41	0.02	0.11
	Central rind	0.81	< LOD	< LOD
	Peripheral rind	0.42	< LOD	< LOD

*^∗^LOD and LOQ were estimated at 0.003 and 0.010 ng/g, respectively. ^∗∗^LOD and LOQ were estimated at 0.013 and 0.035 ng/g, respectively.*

## Discussion and Conclusion

The presence of SEs in any foodstuff represents a potential hazard for human health, under the definition of Article 14 of Regulation (EC) No. 178/2002. Absence of SEs in 5 sample units of 25g has been set as food safety criteria in cheese, by Commission Regulation (EC) No. 2073/2005, amended by Commission Regulation (EC) No. 1441/2007 (Ostyn et al., 2012).

Seven cheese wheels, found positive during own check sampling for CPS presence, with counts exceeding 10ˆ5 cfu/g, were used to study the distribution of microorganisms and enterotoxins within the matrix.

The presence of CPS, performed in the second step of the study, was confirmed in 82% (*n* = 23) of the samples analyzed (*n* = 28), with a concentration ranged between values <1 and 3.7 Log ufc/g in the core samples and between 0.3 and 6.9 Log cfu/g in the rind samples. These values demonstrated that the amount of toxigenic CPS in the milk was enough for SEs production, as SEA and SED were detected in all samples. Even if these results were considered qualitative, the TV and AU values indicated that SEs were present at high concentration level and this was confirmed by the quantification analysis. The amount of SED in all the samples was higher than SEA. In fact, SEA was detected in all wheels of cheese with a concentration between 0.02 to 1.78 ng/g, while SED was quantified in 6 out of 7 wheel (from 0.47 to 3.19 ng/g). The highest concentrations of toxins were found in the wheels 4, while in the wheel 7 it was possible to verify the presence only of SEA.

In total, were identified 50 *S. aureus* isolates among the 7 wheels and 41 were found to have at least one gene able to encode for SE, while 9 were negative. The biotyping results combined with the m-PCR for the identification of SE-encoding genes, highlighted ten different *S. aureus* profiles (Table 4), demonstrating the diversity of the *S. aureus* population structure among seven different cheese productions. The data obtained show the variability of SEs concentrations between the 7 wheels of cheese belonging to different batches and also within the same wheel. It should also be considered that due to the artisanal production, there is no standardization of the final products and that small variations during the production process could have influenced the final concentration of the SEs. Enterotoxin expression is coordinated by a complex network of regulatory elements. Among them, the agr regulatory system plays a crucial role. The agr system positively regulates the expression of many virulence genes (including some genes for enterotoxins: seb, sed, and, presumably, sec), and this expression increases simultaneously with increasing cell density (Valihrach et al., 2014). The study of Duquenne et al. (2016) compared the relative expression levels of the genes *sea*, *seb*, *sec*, and sed in *S. aureus* strains during the manufacture of heat-untreated semi-hard cheeses, with *sed* shown to have the highest expression. While this observation was confirmed by our results, no correlation between the CPS counts and the SEs concentrations was found, when investigating the four areas sampled.

A further factor to take in account is the possible presence of gene variants with different expression rates. A recent study (Johler et al., 2016) demonstrates the presence of different sed gene variant sequences with different enterotoxin expression, highlighting the importance of strain-specific differences and host-specific variation in enterotoxin sequences. The difference observed in the distribution of CPS in the four areas sampled in the second step of the study, is irrespective of the batch and the time of ripening. This is actually in accordance with the previous literature data. Several studies in fact, demonstrated that cheese wheel portions under the rind show a wider biodiversity in the microbiota (Pasquale et al., 2016) and higher bacterial density (Ercolini et al., 2003; Jeanson et al., 2011; Fleurot et al., 2014).

In the present study, however, SEs distribution in the cheese matrix resulted affected by the core versus rind effect, both in the second step of the study, where all the batches were analyzed and the third one, where batch 4 and 7 have been further sub-sampled. This is in contrast with previous findings (Fleurot et al., 2014) where SEs expression was correlated to the cell density. The discrepancy might be due to several factors: firstly, the study conducted by Fleurot et al. analyzed the cheese after 15 days of ripening, while in the present study, the first batch had already 21 days of ripening; secondly, experimental conditions, under which the previous study was conducted, imply that no SEs were present before the cheese making, while in the present study, carried out under “natural” conditions, SEs were likely present in the raw milk. Hence, on one hand, the bacterial density changes in the first 21 days are not known and might have affected the SEs distribution at the time of the analysis; on the other hand, the fact that SEs were likely present before the cheese making, might explain the absence of correlation between cell density and SEs quantity. The core versus rind effect, however, instead of an even distribution, which might have been expected based on the previous speculations, is more difficult to explain. Natural conditions, again, introducing a number of factors such as strain and microbial community variation, might play a role. Even though SEs are known to be resistant to many factors, including proteases (Bhatia and Zahoor, 2007), recent studies (Fujikawa et al., 2017), demonstrated the possibility of SEs degradation by a protease produced by *Pseudomonas* spp. The metabolic activity of the microbial community near the surface, might influence both the persistency and the possibility to detect the SEs.

Further studies should be conducted in order to clarify the reasons of the heterogeneity of CPS counts and SEs distribution and evaluate its impact on measurement uncertainty, so as to identify the best sub-sampling strategy to be used when receiving cheeses for CPS and SE analysis at relevant laboratories. The results of this study provided a set of previously unknown information on the influence of natural conditions on the distribution of CPS and SEs thereof in the cheese matrix. These observations raise a number of interesting questions regarding the influencing factors underlying the phenomenon. In order to understand if the inevitable bias introduced by the “natural conditions” are significant or not, further studies on CPS and SEs distribution will be designed according to the results of this study and carried out under controlled conditions.

## Author Contributions

YN, J-AH, SG, LD, and AB designed the experiments. AB and DB performed the sampling. AB, GM, IM, and SM performed the analyses. AB, FC, GM, YN, and DMB analyzed the data. FC, AB, and GM wrote the manuscript.

## Conflict of Interest Statement

The reviewer VB declared a past collaboration with the authors AB and LD to the handling Editor. The remaining authors declare that the research was conducted in the absence of any commercial or financial relationships that could be construed as a potential conflict of interest.

## References

[B1] Armani M. Macori G. Gallina S. Tavella A. Giusti M. Paolazzi G. (2016). Coagulase positive staphylococci and food poisoning toxins-A case study of an outbreak investigation occurred in a shepherd hut. *Int. J. Infect. Dis.* 45:464 10.1016/j.ijid.2016.02.983

[B2] Bertuzzi A. S. Walsh A. M. Sheehan J. J. Cotter P. D. Crispie F. McSweeney P. L. (2018). Omics-based insights into flavor development and microbial succession within surface-ripened cheese. *MSystems* 3 e211–e217. 10.1128/mSystems.00211-17 29404426 PMC5790873

[B3] Beuchat L. R. (2017). “Influence of water activity on sporulation, germination, outgrowth, and toxin production,” in *Water Activity: Theory and Applications to Food*, eds Rockland L. B. Beuchat L. R. (Abingdon: Routledge),137–151.

[B4] Bhatia A. Zahoor S. (2007). *Staphylococcus aureus* enterotoxins: a review. *J. Clin. Diagn. Res.* 3 188–197. 27348003

[B5] Bianchi D. M. Gallina S. Bellio A. Chiesa F. Civera T. Decastelli L. (2014). Enterotoxin gene profiles of *Staphylococcus aureus* isolated from milk and dairy products in Italy. *Lett. Appl. Microbiol.* 58 190–196. 10.1111/lam.12182 24151939

[B6] Bonham K. S. Wolfe B. E. Dutton R. J. (2017). Extensive horizontal gene transfer in cheese-associated bacteria. *eLife* 6:e22144. 10.7554/eLife.22144 28644126 PMC5526665

[B7] Bueno E. García P. Martínez B. Rodríguez A. (2012). Phage inactivation of *Staphylococcus aureus* in fresh and hard-type cheeses. *Int. J. Food Microbiol.* 158 23–27. 10.1016/j.ijfoodmicro.2012.06.012 22795798

[B8] Cotter P. D. Fox P. F. Everett D. W. McSweeney P. L. (2017). *Cheese: Chemistry, Physics and Microbiology*. Amsterdam: Elsevier Science.

[B9] Devriese L. A. (1984). A simplified system for biotyping *Staphylococcus aureus* strains isolated from different animal species. *J. Appl. Bacteriol.* 56 215–220. 10.1111/j.1365-2672.1984.tb01341.x 6373707

[B10] Duquenne M. Derzelle S. Fleurot I. Aigle M. Darrigo C. Hennekinne J.-A. (2016). Milk maturation temperature and time are key technological parameters to limit staphylococcal enterotoxin production during uncooked semi-hard cheese manufacture. *Food Control* 59 118–127. 10.1016/j.foodcont.2015.05.003

[B11] Ercolini D. Hill P. J. Dodd C. E. R. (2003). Bacterial community structure and location in stilton cheese. *Appl. Environ. Microbiol.* 69 3540–3548. 10.1128/AEM.69.6.3540-3548.2003 12788761 PMC161494

[B12] European Commission [EC] (2005). Commission regulation (EC) No 2073/2005 of 15 November 2005 on microbiological criteria for foodstuffs. *Off. J. Eur. Union* 50 1–26.

[B13] European Commission [EC] (2007). Commission regulation on microbiological criteria for foodstuffs. *Off. J. Eur. Union* 32 12–29.

[B14] European Food Safety Authority [EFSA] and European Centre for Disease Prevention and Control [ECDC] (2015). The European union summary report on trends and sources of zoonoses, zoonotic agents and food-borne outbreaks in 2013. *EFSA J.* 13:3991 10.2903/j.efsa.2015.3991 PMC11926762 40125543

[B15] Fitzsimons N. A. Cogan T. M. Condon S. Beresford T. (2001). Spatial and temporal distribution of non-starter lactic acid bacteria in Cheddar cheese. *J. Appl. Microbiol.* 90 600–608. 10.1046/j.1365-2672.2001.01285.x 11309072

[B16] Fleurot I. Aigle M. Fleurot R. Darrigo C. Hennekinne J.-A. Gruss A. (2014). Following pathogen development and gene expression in a food ecosystem: the case of a *Staphylococcus aureus* isolate in cheese. *Appl. Environ. Microbiol.* 80 5106–5115. 10.1128/AEM.01042-14 24928871 PMC4135746

[B17] Fujikawa H. Nagaoka K. Arai K. (2017). Degradation of staphylococcal enterotoxin A by a *Pseudomonas aeruginosa* isolate from raw milk. *Biosci. Biotechnol. Biochem.* 81 1436–1443. 10.1080/09168451.2017.1314755 28417705

[B18] Hennekinne J. De Buyser M. Dragacci S. (2012). *Staphylococcus aureus* and its food poisoning toxins: characterization and outbreak investigation. *FEMS Microbiol. Rev.* 36 815–836. 10.1111/j.1574-6976.2011.00311.x 22091892

[B19] International Standardization for Organization [ISO] (1999). *Microbiology of Food and Animal Feeding Stuffs—Horizontal Method for the Enumeration of Coagulase-Positive Staphylococci (Staphylococcus aureus and Other Species)—Part 2: Technique Using Rabbit Plasma Fibrinogen Agar Medium. ISO Norm 6888-2:1999*. Geneva: International Standardization for Organization.

[B20] Jablonski L. M. Bohach G. A. (1997). “*Staphylococcus aureus*,” in *Food Microbiology Fundamentals and Frontiers*, eds Doyle M. P. Beuchat L. R. Montville T. J. (Washington, DC: American Society for Microbiology), 353–375.

[B21] Jeanson S. Chadśuf J. Madec M. N. Aly S. Floury J. Brocklehurst T. F. (2011). Spatial distribution of bacterial colonies in a model cheese. *Appl. Environ. Microbiol.* 77 1493–1500. 10.1128/AEM.02233-10 21169438 PMC3067236

[B22] Johler S. Macori G. Bellio A. Acutis P. L. Gallina S. Decastelli L. (2018). *Short communication*: characterization of *Staphylococcus aureus* isolated along the raw milk cheese production process in artisan dairies in Italy. *J. Dairy Sci.* 101 2915–2920. 10.3168/jds.2017-13815 29397175

[B23] Johler S. Sihto H.-M. Macori G. Stephan R. Johler S. Sihto H.-M. (2016). Sequence variability in staphylococcal enterotoxin genes seb, sec, and sed. *Toxins* 8:169. 10.3390/toxins8060169 27258311 PMC4926136

[B24] Johler S. Weder D. Bridy C. Huguenin M. C. Robert L. Hummerjohann J. (2015). Outbreak of staphylococcal food poisoning among children and staff at a Swiss boarding school due to soft cheese made from raw milk. *J. Dairy Sci.* 98 2944–2948. 10.3168/jds.2014-9123 25726108

[B25] Jørgensen H. J. Mørk T. Rørvik L. M. (2005). The occurrence of *Staphylococcus aureus* on a farm with small-scale production of raw milk cheese. *J. Dairy Sci.* 88 3810–3817. 10.3168/jds.S0022-0302(05)73066-6 16230686

[B26] Kérouanton A. Hennekinne J. Letertre C. Petit L. Chesneau O. Brisabois A. (2007). Characterization of *Staphylococcus aureus* strains associated with food poisoning outbreaks in France. *Int. J. Food Microbiol.* 115 369–375. 10.1016/j.ijfoodmicro.2006.10.050 17306397

[B27] Kümmel J. Stessl B. Gonano M. Walcher G. Bereuter O. Fricker M. (2016). *Staphylococcus aureus* entrance into the dairy chain: tracking S. aureus from dairy cow to cheese. *Front. Microbiol.* 7:1603. 10.3389/fmicb.2016.01603 27790200 PMC5061776

[B28] Le Loir Y. Baron F. Gautier M. (2003). *Staphylococcus aureus* and food poisoning. *Genet. Mol. Res.* 2 63–76. 12917803

[B29] Levante A. De Filippis F. La Storia A. Gatti M. Neviani E. Ercolini D. (2017). Metabolic gene-targeted monitoring of non-starter lactic acid bacteria during cheese ripening. *Int. J. Food Microbiol.* 257 276–284. 10.1016/j.ijfoodmicro.2017.07.002 28735145

[B30] Macori G. Cotter P. D. (2018). Novel insights into the microbiology of fermented dairy foods. *Curr. Opin. Biotechnol.* 49 172–178. 10.1016/j.copbio.2017.09.002 28964915

[B31] McHugh A. J. Feehily C. Hill C. Cotter P. D. (2017). Detection and enumeration of spore-forming bacteria in powdered dairy products. *Front. Microbiol.* 8:109. 10.3389/fmicb.2017.00109 28197144 PMC5281614

[B32] Nia Y. Mutel I. Assere A. Lombard B. Auvray F. Hennekinne J. A. (2016). Review over a 3-year period of European Union proficiency tests for detection of staphylococcal enterotoxins in food matrices. *Toxins* 8:107. 10.3390/toxins8040107 27089364 PMC4848633

[B33] Ostyn A. Buyser M. Guillier F. Krys S. Hennekinne J. (2012). Benefits of the combined use of immunological- and PCR-based methods for determination of staphylococcal enterotoxin food safety criteria in cheeses. *Food Anal. Methods* 5 173–178. 10.1007/s12161-011-9244-y

[B34] Pasquale I. D. Cagno R. D. Buchin S. Angelis M. D. Gobbetti M. (2016). Spatial distribution of the metabolically active microbiota within Italian PDO ewes’ milk cheeses. *PLoS One* 11:e0153213. 10.1371/journal.pone.0153213 27073835 PMC4830609

[B35] Schelin J. Wallin-Carlquist N. Thorup Cohn M. Lindqvist R. Barker G. C. (2011). The formation of *Staphylococcus aureus* enterotoxin in food environments and advances in risk assessment. *Virulence* 2 580–592. 10.4161/viru.2.6.18122 22030860 PMC3260550

[B36] Sihto H. M. Tasara T. Stephan R. Johler S. (2014). Validation of reference genes for normalization of qPCR mRNA expression levels in *Staphylococcus aureus* exposed to osmotic and lactic acid stress conditions encountered during food production and preservation. *FEMS Microbiol. Lett.* 356 134–140. 10.1111/1574-6968.12491 24893820

[B37] Valihrach L. Alibayov B. Zdenkova K. Demnerova K. (2014). Expression and production of staphylococcal enterotoxin C is substantially reduced in milk. *Food Microbiol.* 44 54–59. 10.1016/j.fm.2014.05.020 25084645

[B38] Wolfe B. E. Button J. E. Santarelli M. Dutton R. J. (2014). Cheese rind communities provide tractable systems for in situ and in vitro studies of microbial diversity. *Cell* 158 422–433. 10.1016/j.cell.2014.05.041 25036636 PMC4222527

